# Matrix-Assisted Laser Desorption/Ionization Time-of-Flight Mass Spectrometry Identification of Mycobacteria in Routine Clinical Practice

**DOI:** 10.1371/journal.pone.0024720

**Published:** 2011-09-13

**Authors:** Amel El Khéchine, Carine Couderc, Christophe Flaudrops, Didier Raoult, Michel Drancourt

**Affiliations:** URMITE UMR CNRS 6236 IRD198, Institut Hospitalier Universitaire POLMIT, IFR48, Université de la Méditerranée et Pôle de Maladies Infectieuses, Assistance Publique-Hôpitaux de Marseille, Marseille, France; Charité, Campus Benjamin Franklin, Germany

## Abstract

**Background:**

Non-tuberculous mycobacteria recovered from respiratory tract specimens are emerging confounder organisms for the laboratory diagnosis of tuberculosis worldwide. There is an urgent need for new techniques to rapidly identify mycobacteria isolated in clinical practice. Matrix-assisted laser desorption time-of-flight mass spectrometry (MALDI-TOF MS) has previously been proven to effectively identify mycobacteria grown in high-concentration inocula from collections. However, a thorough evaluation of its use in routine laboratory practice has not been performed.

**Methodology:**

We set up an original protocol for the MALDI-TOF MS identification of heat-inactivated mycobacteria after dissociation in Tween-20, mechanical breaking of the cell wall and protein extraction with formic acid and acetonitrile. By applying this protocol to as few as 10^5^ colony-forming units of reference isolates of *Mycobacterium tuberculosis*, *Mycobacterium avium*, and 20 other *Mycobacterium* species, we obtained species-specific mass spectra for the creation of a local database. Using this database, our protocol enabled the identification by MALDI-TOF MS of 87 *M. tuberculosis*, 25 *M. avium* and 12 non-tuberculosis clinical isolates with identification scores ≥2 within 2.5 hours.

**Conclusions:**

Our data indicate that MALDI-TOF MS can be used as a first-line method for the routine identification of heat-inactivated mycobacteria. MALDI-TOF MS is an attractive method for implementation in clinical microbiology laboratories in both developed and developing countries.

## Introduction

Pulmonary tuberculosis is a deadly, contagious infection caused by mycobacteria belonging to the *Mycobacterium tuberculosis* complex (MTC) [1]. Tuberculosis remains in the top 10 health priorities in many developing countries, and with more than 1.3 million new cases yearly [2] and the emergence and rapid spread of multidrug-resistant and extensively drug-resistant *M. tuberculosis* strains in such countries, the rapid laboratory diagnosis of tuberculosis is essential. However, this task has been complicated by the emergence of non-tuberculous mycobacteria present in respiratory tract specimens from patients suspected of pulmonary tuberculosis, even in countries with a high incidence and burden of pulmonary tuberculosis [3]. For example, a recent study showed that 52.5% of non-tuberculous isolates in Taiwan were from respiratory tract specimens [4]. Indeed, the actinobacterial genus *Mycobacterium* comprises more than 120 species including both obligate human pathogens, such as the leprosy agent *Mycobacterium leprae* [5], and the MTC members and opportunistic pathogens, such as the *Mycobacterium avium* complex and the *Mycobacterium abscessus* group mycobacteria and harmless environmental organisms [6].

The identification of mycobacteria has long been based on conventional phenotypic methods requiring extensive incubation times and delaying the final diagnosis far beyond medical recommendations [3]. In some developing countries, these methods have been successfully replaced by the direct microscopic detection of cords specific for *M. tuberculosis* after a 5–10-day incubation of the specimen in either agar or miniaturized liquid medium [7], [8]. However, these techniques still require some degree of expertise and rely on microscopic examination, an operator-dependent method that is prone to errors. While some other techniques for the phenotypic analysis of mycobacteria, such as the high-pressure liquid chromatography of cell wall mycolic acids, proved efficient at identification, they have not been implemented as a routine technique in clinical microbiology laboratories [9], [10]. Molecular methods including PCR-based hybridization and sequencing methods [11], [12] are now routinely used for the identification of mycobacteria in laboratories in developed countries. Recently, confined real-time PCR has been shown to be effective for the rapid detection of tuberculosis, including rifampin-resistant tuberculosis, in respiratory tract specimens [13], [14]. These molecular techniques still require specific technical expertise and technically demanding, expensive equipment [15]–[17].

Several studies have provided the proof-of-concept that matrix-assisted laser desorption ionization time-of-flight mass spectrometry (MALDI-TOF MS) could also identify mycobacteria [18]–[21]. Indeed, this technique has emerged over the last few years as a revolutionary technique for the routine identification of bacterial isolates [22]–[32]. MALDI-TOF MS has been shown to yield accurate results within hours at a lower price than any other method routinely used in clinical microbiology laboratories [32]. However, the studies supporting this method analyzed high-concentration inocula [18]–[21]. In a few studies, mycobacteria were analyzed without previous inactivation [21], [33], which does not comply with current standards for the manipulation of harmful organisms.

We therefore aimed to further explore various inactivation protocols and solvents that one might consider for mycobacteria with the perspective to decrease the inoculum required for the accurate MALDI-TOF MS identification of mycobacteria, thus speeding the diagnosis of tuberculosis and other non-tuberculosis, mycobacterial infections.

## Methods

### *Mycobacterium* strains

As a first step, we used 11 strains of mycobacteria representative of 7 *M. tuberculosis* complex species, 12 strains of mycobacteria representative of 11 *M. avium* complex species and 20 additional mycobacteria strains representative of 16 additional non-tuberculous species grown from our collection to set up a reference database (Table 1). In a second step, all the mycobacteria isolated from February 2010 to May 2011 from patients in the Mycobacteria Reference Laboratory, Institut Hospitalier Universitaire POLMIT, Marseilles, France were prospectively analyzed by MALDI-TOF MS. The isolates were cultured in MGIT tubes (Becton Dickinson, Pont-De-Claix, France) or on a home-made 5% sheep blood-agar (BioMérieux, La Balme-les-Grottes, France) for 2–45 days at 32°C or 37°C as previously described [34], [35]. Gram staining was used to ensure the absence of any contaminant organisms in the culture, and standard identification of mycobacteria was performed by observing acid-fast organisms after Ziehl-Neelsen staining, *rpoB* sequencing for non-tuberculosis mycobacteria [36] and exact tandem repeat D sequencing (ETR-D) for MTC [12]. We tested two protocols for the inactivation of mycobacteria before the MALDI-TOF MS by incubating 1 mL of the mycobacterial suspension obtained by suspending colonies from solid medium or 3 mL of mycobacteria suspension of liquid medium either in a water bath at 95°C for one hour (Table 2, protocol A) [37] or in 70% ethanol for 10 minutes (Table 2, protocol B) [19]. The effectiveness of each inactivation protocol was tested in triplicate by inoculating 50 µl inactivated *M. tuberculosis* H37Rv, *M. bovis* BCG and *M. fortuitum* suspensions in MGIT tubes incubated in parallel at 37°C. The absence of any visible growth after incubation for 45 days was taken as an evidence for the effective inactivation of the mycobacteria.

**Table 1:** Reference mycobacteria strains used to create a MALDI-TOF MS database for the identification of mycobacteria.

*Mycobacterium tuberculosis* complex	
*M. tuberculosis*	CIP 103471; H37Rv; CH6431
*M. africanum*	CIP 105147
*M. bovis*	CIP 671203; CIP 108541^T^; CIP105050
*M. bovis* BCG Tokyo	ATCC 35737
*M. caprae*	CIP105776
*M. microti*	CRBIP7.40
*M. canettii*	CIP 140010059^T^
*M. pinnipedii*	ATCC-BAA-688

*Mycobacterium avium* complex	
*M. avium* subsp. *hominissuis*	IWGMT49
*M. avium* subsp. *avium*	CIP104244^T^
*M. avium* subsp. *paratuberculosis*	CIP103963
*M. avium* subsp. *silvaticum*	CIP103317
*M. colombiense*	FI 04062
*M. chimaera*	CIP 107892^T^; DSM 446232
*M. marseillense*	CIP 109828^T^
*M. timonense*	CIP 109830^T^
*M. bouchedurhonense*	CIP 109827^T^
*M. intracellulare*	CIP104243^T^
*M. indicus pranii* *	DSM 45239*
*M. arosiense*	DSM 45069^T^

Other Mycobacteria	
*M. smegmatis*	CIP104444^T^, ATCC 700084
*M. abscessus* subsp. *abscessus*	DSM 43567, DSM 43435, DSM 43439, CIP104536^T^
*M. abscessus* subsp. *bolletii*	CIP108541^T^, CIP108297
*M. aurum*	CIP104465^T^
*M. chelonae*	CIP104535^T^
*M. fortuitum* subsp. *fortuitum*	CIP104524^T^
*M. gordonae*	CIP104529^T^
*M. kansasii*	CIP104589^T^
*M. marinum*	CIP104528^T^
*M. phlei*	CIP105389^T^
*M. xenopi*	CIP104035^T^
*M. immunogenum*	CIP 106684^T^
*M. lentiflavum*	CIP105465
*M. simiae*	CIP104531^T^

*M. indicus pranii **is not a validated species.**

**Table 2:** Inactivation protocols and solvents used to treat mycobacteria for identification by MALDI-TOF MS.

Protocol	Inactivation protocol	
A	95°C/60 minutes	
B	70% ethanol/10 minutes	

	Solvent type and composition	
1	0.5% Tween-20	Mycobacteria aggregate dissociation
2	5% β-mercaptoethanol	Mycobacteria aggregate dissociation
3	Acetone	Washing
4	Absolute ethanol	Protein precipitation
5	0.1 M NaOH	Protein extraction/elution
6	10% TFA	Protein extraction/elution
7	0.1 M HCl	Protein extraction/elution
8	70% HCOOH	Protein extraction/elution

### MALDI-TOF MS protocol for mycobacteria

Several protocols were tested before finalizing the protocol for the preparation of inactivated mycobacteria. Because some mycobacteria, including the MTC mycobacteria, are known to form cords which may hamper the MALDI-TOF MS analysis, we tested several compounds for their ability to solubilize such aggregates. We tested the incubation of mycobacteria in a detergent solution of 0.5% Tween-20 (Table 2, protocol 1) or β-mercaptoethanol, a chemical known to reduce disulfide bonds in proteins [38] (Table 2, protocol 2). We also tested the effects of using acetone (Table 2, protocol 3) or distilled water as a washing solution; absolute ethanol for protein precipitation (Table 2, protocol 4); and 0.1 M NaOH (Table 2, protocol 5), 10% **v/v** trifluoroacetic acid (TFA) (Table 2, protocol 6), 0.1 M HCl (Table 2, protocol 7) or 70% formic acid for protein elution (Table 2, protocol 8). Based on the results of these preliminary tests, protocol 8 was used for the final experiments. For each parameter, we compared the results obtained with *M. tuberculosis* H37Rv, *M. bovis* BCG and *Mycobacterium fortuitum* with the definitive reference profile, in terms of identification score (from 0 to 3), number of peaks in the 5,000–20,000 m/z range, and the intensity of the peaks, as determined by the Flex Analysis 3.0 software (Bruker Daltonics). Each test was done ten times.

### Sensitivity of MALDI-TOF MS detection

In order to determine the sensitivity of our final protocol, serial 10-fold dilutions from 10^8^ to 10^3^ mycobacteria/mL were prepared for *M. tuberculosis* H37Rv, *M. bovis* BCG and *Mycobacterium fortuitum*. For each suspension, the inoculum was calibrated using two methods. First, by counting the mean number of mycobacteria observed in five areas on three slides of Ziehl-Neelsen stained mycobacteria observed at a 1,000× magnification. Second, by culturing the dilutions on blood agar plates for 5 weeks and counting the number of colonies. In order to facilitate this quantification, ImageJ colony-counting software (http://rsb.info.nih.gov/ij/) was used. The sensitivity was then calculated by reporting the colony number with the fold dilutions.

### Mycobacterial MALDI-TOF database

To complement the Bruker Daltonics database, we analyzed a collection of reference strains for mycobacteria using protocol 8, as presented below. For each reference mycobacterial strain, one colony was selected from the agar plate for the solid medium, and approximately 3 ml of mycobacterial suspension was extracted for the liquid medium and was manipulated as described above. Each reference strain was analyzed four times using the same protocol. For each sample, 1.5 µL was deposited on a polished steel MSP 96 target plate (Bruker Daltonics, Bremen, Germany) and 1.5 µL of matrix solution (saturated α-cyano-4-hydroxycinnamic acid, 50% acetonitrile, 2.5% trifluoroacetic acid) was then added. The samples were air-dried for 5 minutes before being processed in the mass spectrometer. To validate the analysis of a whole MSP 96 target, Bacterial Test Standard (*Escherichia coli* DH5alpha protein extract; Bruker Daltonics ref 255343) was used as a positive control and non-inoculated matrix solution (alpha-cyano-4-Hydroxycinnamic acid) was used as a negative control on each plate. The analyses were performed using the Microflex MALDI-TOF MS spectrometer (Bruker Daltonics) at 337 nm with the Flex control software (Bruker Daltonics). Positive ions were extracted with an accelerating voltage of 20 KV in linear mode. The spectra were analyzed within an m/z range of 2,000 to 20,000. Four raw spectra were automatically acquired using the Flexcontrol 3.0 software and then compared with the Bruker Daltonics database using the MALDI Biotyper 2.0 Bruker Daltonics software. To validate the analysis using the MALDI Biotyper software, the identification of the positive control was required to be *E. coli* with an identification score ≥2, and the negative control had to yield a non-identifying score ≤1.7.

The profiles of the 11 reference strains for the *M. tuberculosis* complex, 12 reference strains for the *M. avium* complex and 20 reference strains for the other non-tuberculous mycobacteria were compared and analyzed using the Biotyper 2.0 software, and the MALDI-TOF MS spectra were entered into a local *Mycobacterium* database that was further merged with the 3,438-entry Bruker Daltonics database (June 2010).

### MALDI-TOF MS identification of mycobacteria

All the clinical *Mycobacterium* isolates prospectively obtained from the Mycobacteria Reference Laboratory, Institut Hospitalier Universitaire POLMIT, Marseilles, France from February 2010 to May 2011 were prospectively analyzed using protocol 8, as presented below. To identify each clinical isolate, the corresponding spectra were then compared with those in the local *Mycobacterium* database merged with the Bruker Daltonics database using the Biotyper software 2.0.

## Results

### MALDI-TOF MS protocol for mycobacteria

**Figure 1:**
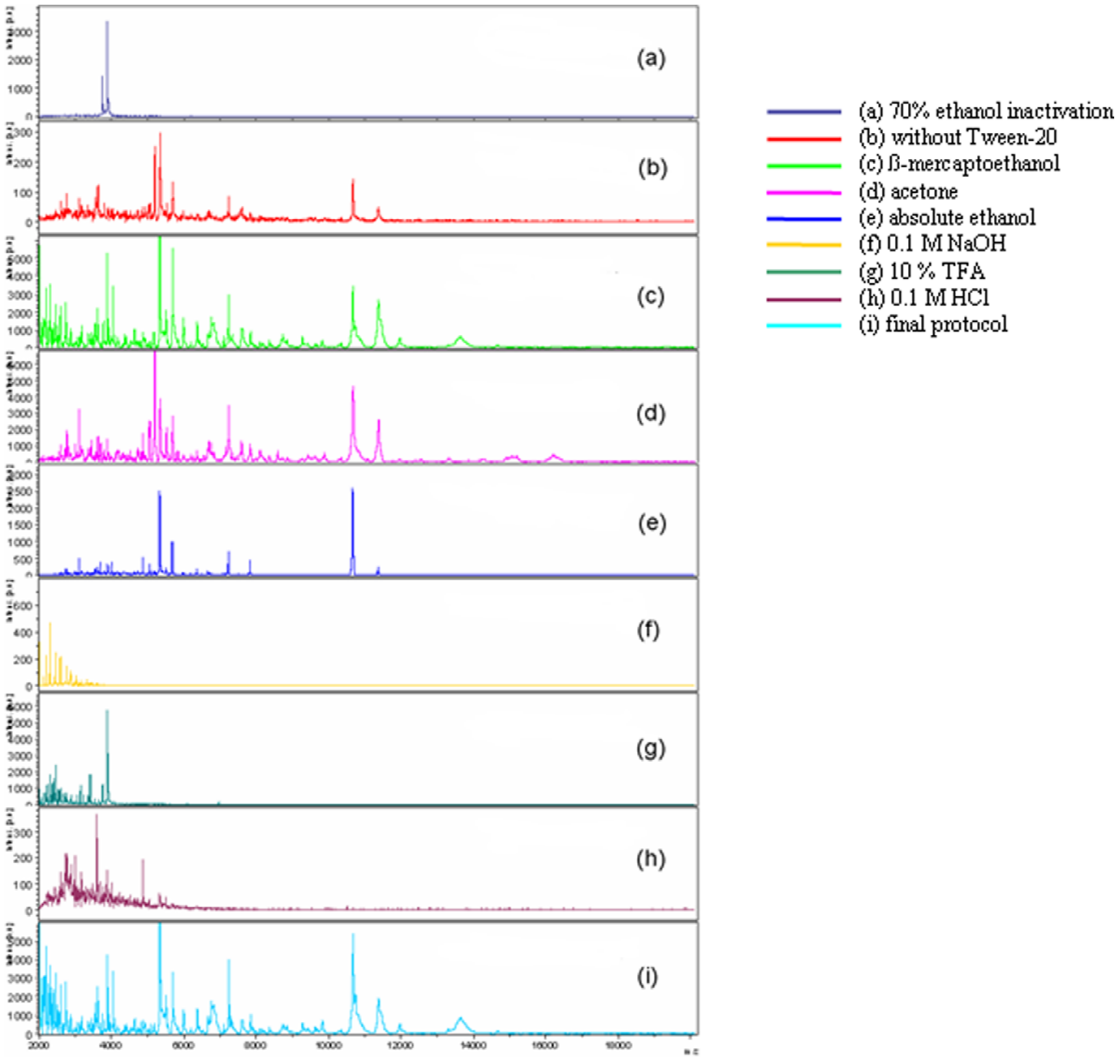
MALDI-TOF MS spectra obtained from the *M. tuberculosis* H37Rv strain using the protocols tested herein. Protocol A was used throughout; protocol B was used where mentioned (Line 2).

We observed no growth of *M. tuberculosis*, *M. bovis* BCG and *M. fortuitum* mycobacteria over a 45-day incubation after either heat inactivation or a 10-minute incubation with 70% ethanol. However, the incubation of Middlebrook 7H9-grown mycobacteria in ethanol yielded protein profiles of lower quality (Figure 1 part a) than those obtained after heat-inactivation. Therefore, the 1-hour incubation at 95°C was incorporated into our final protocol. Likewise, because incubating mycobacteria in 0.5% Tween-20 yielded spectra of better quality than mycobacteria run in parallel without Tween-20 treatment (Table 3.1; Figure 1 part b), 0.5% Tween-20 treatment was incorporated into our final protocol. We found that the suspension of mycobacteria in β-mercaptoethanol did not improve the quality of spectra (Table 3.2; Figure 1 part c), so β-mercaptoethanol was not used in our final protocol. We then tested the use of acetone and distilled water as washing solutions. No difference was observed between the spectra; both resulted in adequate identification with almost the same intensity and score (Table 3.3; Figure 1 part d). Acetone took longer to manipulate and to dry than distilled water, which was therefore retained in the final protocol. We observed that protein precipitation using absolute ethanol (Table 3.4; Figure 1 part e) did not improve the quality of the spectra when compared with distilled water alone. We also tested several protein extraction and elution solvents. No peak was observed in the 5,000–15,000 Kda range with 0.1 M NaOH (Table 3.5; Figure 1 part f) or 0.1 M HCl (Table 3.6; Figure 1 part.f), only one peak was observed with 10% TFA (Table 3.7; Figure 1 part g) and 11 peaks were obtained using 70% HCOOH (Table 3.8; Figure 1 part i). We therefore incorporated 70% HCOOH combined with 100% acetonitrile into our final protocol.

**Table 3:** Spectra obtained from *Mycobacterium tuberculosis* H37Rv using the tested protocols.

Protocol	Number of peaks	Mean intensity (+/− standard deviation) of the most prevalent peaks						Identification score average
		5340 m/z	5500 m/z	5690 m/z	10670 m/z	10700 m/z	11380 m/z	
**(1)**	8	270(+/−78)	171(+/−57)	139(+/−45)	118(+/−37)	61(+/−14)	44(+/−12)	1.45
**(2)**	8	5846(+/−2797)	1804(+/−1007)	2544(+/−1248)	1968(+/−960)	1400(+/−594)	1518(+/−213)	1.87
**(3)**	11	5349(+/−4850)	4037(+/−3835)	4314(+/−2912)	2847(+/−2851)	2077(+/−2115)	2557(+/−2229)	2.17
**(4)**	11	4798(+/−5400)	2531(+/−2324)	1950(+/−2252)	1681(+/−1709)	949(+/−1258)	675(+/−1380)	2.43
**(5)**	0	−						−
**(6)**	0	−						−
**(7)**	1	1007 (+/− 260)						−
**(8)**	11	6166(+/−2477)	2910(+/−2503)	3122(+/−2212)	2276(+/−1580)	2435(+/−1539)	2148(+/−1708)	2.51

Tests were done 10 times by using inactivation protocol A.

Protocol 8 (Table 3.9; Figure 1 part i) was used as the final protocol. Colonies from each isolate were collected in a screw-cap Eppendorf tube containing 500 µl of HPLC-grade water and 0.5% Tween-20 and inactivated by heating at 95°C for one hour [37]. The mycobacterial suspensions were then washed twice with 500 µL of HPLC-grade water and centrifuged at 13,000×g for 10 min. The pellet was vortexed along with 500 µL of HPLC-grade water and 0.3 g acid-washed glass beads (diameter ≤106 µm, Sigma) in a BIO 101 FastPrep instrument (Qbiogene, Strasbourg, France) at level 6.5 (full speed) for 3 min. The suspension was centrifuged at 13,000×g for 10 min. The pellet was then resuspended in 5 to 50 µl 70% HCOOH and 5 to 50 µl 100% acetonitrile, depending on the volume of the pellet. The suspension was centrifuged at 11,000×g for 1 min and 1.5 µl of the supernatant was deposited on a target plate (Bruker Daltonics, Bremen, Germany) in four replicates. Finally, 1.5 µl matrix solution (saturated α cyano-4-hydroxycinnamic acid, 50% acetonitrile, 2.5% TFA) was added and allowed to co-crystallize with the sample pellet at room temperature.

### Mycobacterium MALDI-TOF database

Using our optimized protocol, the lower limit of detection of MALDI-TOF MS was about 10^5^ CFU/mL, which corresponds to 10^3^ mycobacteria deposited on the MALDI-TOF MS plate. All of the mycobacteria reference strains tested by mass spectrometry had a species-specific spectrum profile. Spectrum profiles were deposited in a spectrum database that is an internal MALDI-TOF database of La Timone hospital Which combined with the Bruker database yielded a total of 141 reference spectra comprising of 62 *Mycobacterium* species (Table S1).

### MALDI-TOF identification of mycobacteria

In all cases, the negative control wells yielded either no visible peaks or faint profiles that were not interpreted by the system, and the positive controls yielded the expected protein profile. A total of 124 clinical isolates were prospectively analyzed within a 16-month period by MALDI-TOF MS and all tested mycobacterial isolates yielded a visible protein profile with an identification score of 2 to 2.7. Of the clinical isolates, 87 were identified as *M. tuberculosis*, 17 as *M. avium* subsp. *hominisuis*, 7 as *M. intracellulare*, one as *M. marsiliense*, 7 *M. chelonae*, 2 as *M. abscessus*, 2 as *M. kansasii* and one as *M. fortuitum*. In all cases, the MALDI-TOF MS identification agreed with the molecular identification routinely performed in our laboratory using either ETR-D sequencing for the identification of MTC species [12] or partial *rpoB* sequencing for the other mycobacteria [36].

## Discussion

The protein spectra obtained from the reference and clinical mycobacterial strains in this study were interpreted as accurate and specific. In each case, the positive controls yielded the expected and identifying profiles, whereas the negative controls yielded either no detectable peaks or faint profiles that were not identified by the system. Moreover, the identity of both the reference strains and the clinical isolates were validated by partial *rpo*B sequencing in parallel with the MALDI-TOF MS identification [36].

In this study, MALDI-TOF MS spectra were obtained after the inactivation of the isolates. *M. tuberculosis* complex mycobacteria are harmful organisms that have previously been found to be responsible for cases of laboratory-acquired tuberculosis in laboratory staff [39]. *M. tuberculosis* species are Biosafety Level 3 organisms that must be inactivated prior to manipulation outside a biological safety cabinet to avoid potentially exposing the laboratory staff to contamination [39], [40]. Because the tuberculous nature of AFB organisms cannot be determined by microscopic observation, we suggest that systematic inactivation of AFB organisms is warranted prior to their identification by MALDI-TOF MS. Although some previous studies did not mention how the mycobacteria were inactivated prior to MALDI-TOF MS analysis [21], [33], ethanol has been used for this purpose [19], [41]. We noticed that the latter protocol relied on the centrifugation of mycobacteria prior to ethanol inactivation, which carries the risk of generating MTC mycobacteria aerosols that are potentially harmful for the laboratory staff. We aimed to avoid any centrifugation of non-inactivated mycobacteria and therefore chose to inactivate the mycobacteria by heating, a procedure previously reported to effectively inactivate mycobacteria, including MTC mycobacteria, after 30 minutes at 95°C [18], [37], [42], [43]. We were also concerned that the use of ethanol could interfere with the quality of the spectra because ethanol has a protein precipitating action. Indeed, when we used ethanol to kill mycobacteria in liquid medium, it precipitated all the proteins in the medium, including those of the mycobacteria. As previously reported [21], the composition of the medium (either Middlebrook 7H9 liquid medium or solid blood-agar medium) did not interfere with identification by mass spectrometry, especially after the incorporation of washing steps.

In this study, the addition of Tween-20 during the inactivation phase increased the quality of the MALDI-TOF MS spectra of mycobacteria. Tween-20 is a detergent that disrupts the cords formed by mycobacteria. Bruker and the authors of a later work [18] used a micropestle for this step. However, we chose to use Tween-20, which is easier to manipulate and more cost-effective. Breaking the cell wall was another crucial step that facilitated the extraction of mycobacterial proteins. Indeed, we previously observed that disrupting the strong cell wall of mycobacteria was necessary for their proper molecular detection [44]. This was also the case for the molecular detection of methanogenic *Archaea*, another group of prokaryotes with a strong cell wall, using either a molecular approach [45] or MALDI-TOF MS (Dridi and Drancourt, unpublished data). Using the protocol described here allowed us to decrease the required inoculum of mycobacteria down to 10^5^ CFU/mL, which is the lowest inoculum reported for the efficient identification of mycobacteria by MALDI-TOF MS. Although the initial studies and some recent reports used a non-calibrated inoculum [18], [19], [21], more recent studies incorporated a high-concentration (10^8^–10^10^ CFU/mL) inoculum [33]. Obtaining such a high-concentration inoculum requires extensive, time-consuming culture and exposes laboratory personnel to potentially harmful pathogens for extended periods of time.

Over the past five years, our Mycobacteriology Reference Laboratory, serving a cosmopolitan area with 1–2 million inhabitants identified 354 new cases of *Mycobacterium* infection, including 295 new cases of *M. tuberculosis* infection and 33 new cases of MAC infections (2005 to 2009). Other non-tuberculous mycobacteria isolated in our laboratory during this period were *M. chelonae* (7 cases), *M. lentiflavum* (5 cases), *M. fortuitum* (3 cases), *M. xenopi* (3 cases), *M. gordonae* (3 cases), *M. kansasii* (2 cases), *M. marinum* (2 cases) and *M. phocaicum* (1 case). We set up a MALDI-TOF MS database that included the *Mycobacterium* organisms most frequently identified over the last several years. This local database perfectly completed the original Bruker Daltonics database, which contained approximately 80 *Mycobacterium* spectra representative of 40 *Mycobacterium* species (June 2010 version) (Table S1). Although this database is enriched in *Mycobacterium* species most frequently encountered in our geographical region, nevertheless it is the largest *Mycobacterium* MALDI-TOF database ever published, comprising of 62 species and 177 references to be compared with previous reports comprising of 4–53 species and 5–53 reference spectra [18]–[21]. Therefore, present database could likely serve as an efficient tool for the identification of mycobacteria in other geographical regions. Although the *M. tuberculosis* complex and *M. avium* complex organisms together represented more than 90% of *Mycobacterium* isolates in our laboratory, the Bruker Daltonics database included only eight *M. tuberculosis* spectra and six *M. avium* spectra out of a total of 40 *Mycobacterium* spectra. In contrast, we previously noted that a library of ≥10 reference spectra was necessary for the accurate MALDI-TOF MS identification of cultured bacteria [25]. In this work, we therefore expanded the Bruker Daltonics database by 37% by adding a total of 43 reference MALDI-TOF MS spectra, and we doubled the number of MTC references and tripled those of MAC.

In this study, we evaluated the prospective use of MALDI-TOF MS for the routine identification of mycobacteria isolates from specimens in patients suspected of pulmonary tuberculosis. While previous studies provided the proof-of-concept that MALDI-TOF MS could be used for the first-line identification of mycobacteria [18], [19], [21], these studies were based on the retrospective analysis of mycobacteria retrieved from collections. The few studies incorporating both *M. tuberculosis* complex isolates and non-tuberculous mycobacteria suffered from limitations that would prevent their application to routine laboratory use.

In conclusion, MALDI-TOF MS has been successfully adapted for the routine identification of mycobacteria. This revolutionary technique allows for the easier and faster diagnosis of mycobacterial pathogens compared with conventional phenotypic identification methods. MALDI-TOF MS therefore appears to be an alternative first-line approach to the routine identification of the vast majority of bacteria commonly cultured in the clinical microbiology laboratory [25], [32].

## Supporting Information

Table S1 List of 62 *Mycobacterium* species included in the MALDI-TOF database. This database combined Bruker and home-made databases. For each species/sub-species, the number of reference spectra is indicated, for a total of 177 references.
(DOC)
