# Supplementary material for: Matrix-Assisted Laser Desorption/Ionization Time-of-Flight Mass Spectrometry Identification of Mycobacteria in Routine Clinical Practice
Source: PLoS One. 2011 Sep 13;6(9):e24720. doi: 10.1371/journal.pone.0024720 (PMC3172293; doi:10.1371/journal.pone.0024720)
Supplement: Table S1 — List of 62 Mycobacterium species included in the MALDI-TOF database. This database combined Bruker and home-made databases. For each species/sub-species, the number of reference spectra is indicated, for a total of 177 references. (DOC) [file pone.0024720.s001.doc]

**Table S1. List of 62 *Mycobacterium* species included in the MALDI-TOF database. This database combined Bruker and home-made databases. For each species/sub-species, the number of reference spectra is indicated, for a total of 177 references.**

| **Species** | **Number of reference spectra** |
| --- | --- |
| ***Mycobacterium tuberculosis* complex** | |
| *M. tuberculosis* | 29 |
| *M. africanum* | 1 |
| *M. bovis* | 4 |
| *M. bovis* BCG | 2 |
| *M. caprae* | 1 |
| *M. microti* | 1 |
| *M. canettii* | 1 |
| *M. pinnipedii* | 1 |
| ***Mycobacterium avium* complex** | |
| *M. avium* subsp. *hominissuis* | 12 |
| *M. avium* subsp. *avium* | 1 |
| *M. avium* subsp. *paratuberculosis* | 2 |
| *M. avium* subsp. *silvaticum* | 1 |
| *M. colombiense* | 1 |
| *M. chimaera* | 2 |
| *M. marseillense* | 1 |
| *M. timonense* | 1 |
| *M. bouchedurhonense* | 1 |
| *M. intracellulare* | 8 |
| *M. indicus pranii******** | 1 |
| *M. arosiense,* | 1 |
| **Other Mycobacteria** | |
| *M. abscessus* subsp. *abscessus* | 8 |
| *M. abscessus* subsp. *bolletii* | 2 |
| *M. agri* | 1 |
| *M. asiaticum* | 1 |
| *M. aurum* | 1 |
| *M. boenicei* | 1 |
| *M. celatum* | 3 |
| *M. chelonae* | 10 |
| *M. chlorophenolicum* | 1 |
| *M. conceptionense* | 1 |
| *M. farcinogenes* | 1 |
| *M. fortuitum* subsp. *fortuitum* | 5 |
| *M. gordonae* | 7 |
| *M. heckeshornense* | 1 |
| *M. hiberniae* | 1 |
| *M. hodleri* | 1 |
| *M. immunogenum* | 1 |
| *M. kansasii* | 8 |
| *M. kumamotonense* | 1 |
| *M. lacus* | 1 |
| *M. lentiflavum* | 1 |
| *M. mageritense* | 1 |
| *M. malmoense* | 3 |
| *M. manitobense* | 1 |
| *M. marinum* | 2 |
| *M. montefiorense* | 1 |
| *M. mucogenicum* | 1 |
| *M. palustre* | 1 |
| *M. peregrinum* | 2 |
| *M. phlei* | 2 |
| *M. pseudoshottsii* | 1 |
| *M. pulveris* | 1 |
| *M. rhodesiae* | 1 |
| *M. seoulense* | 1 |
| *M. shottsii* | 1 |
| *M. simiae* | 4 |
| *M. smegmatis* | 6 |
| *M. szulgai* | 1 |
| *M. thermoresistibile* | 1 |
| *M. tokaiense* | 1 |
| *M. ulcerans* | 1 |
| *M. xenopi* | 5 |

* *M. indicus pranii* is not validated species.
